# Complex Evolutionary History of the *Aeromonas veronii* Group Revealed by Host Interaction and DNA Sequence Data

**DOI:** 10.1371/journal.pone.0016751

**Published:** 2011-02-16

**Authors:** Adam C. Silver, David Williams, Joshua Faucher, Amy J. Horneman, J. Peter Gogarten, Joerg Graf

**Affiliations:** 1 Department of Molecular and Cell Biology, University of Connecticut, Storrs, Connecticut, United States of America; 2 Pathology and Laboratory Medical Services, VA Maryland Health Care System, Baltimore, Maryland, United States of America; 3 Department of Medical and Research Technology, University of Maryland School of Medicine, Baltimore, Maryland, United States of America; J. Craig Venter Institute, United States of America

## Abstract

*Aeromonas veronii* biovar sobria, *Aeromonas veronii* biovar veronii, and *Aeromonas allosaccharophila* are a closely related group of organisms, the *Aeromonas veronii* Group, that inhabit a wide range of host animals as a symbiont or pathogen. In this study, the ability of various strains to colonize the medicinal leech as a model for beneficial symbiosis and to kill wax worm larvae as a model for virulence was determined. Isolates cultured from the leech out-competed other strains in the leech model, while most strains were virulent in the wax worms. Three housekeeping genes, *recA*, *dnaJ* and *gyrB*, the gene encoding chitinase, *chiA*, and four loci associated with the type three secretion system, *ascV*, *ascFG*, *aexT*, and *aexU* were sequenced. The phylogenetic reconstruction failed to produce one consensus tree that was compatible with most of the individual genes. The Approximately Unbiased test and the Genetic Algorithm for Recombination Detection both provided further support for differing evolutionary histories among this group of genes. Two contrasting tests detected recombination within *aexU*, *ascFG*, *ascV*, *dnaJ*, and *gyrB* but not in *aexT* or *chiA*. Quartet decomposition analysis indicated a complex recent evolutionary history for these strains with a high frequency of horizontal gene transfer between several but not among all strains. In this study we demonstrate that at least for some strains, horizontal gene transfer occurs at a sufficient frequency to blur the signal from vertically inherited genes, despite strains being adapted to distinct niches. Simply increasing the number of genes included in the analysis is unlikely to overcome this challenge in organisms that occupy multiple niches and can exchange DNA between strains specialized to different niches. Instead, the detection of genes critical in the adaptation to specific niches may help to reveal the physiological specialization of these strains.

## Introduction

Over 150 years after Darwin published the Origin of Species [1], the mechanisms by which organismal lineages separate and diverge remain an intensely studied problem in evolutionary biology. How do geographical separation, ecological adaptation, and the accumulation of genetic differences cause barriers to gene flow that ultimately lead to distinct lineages? If species can be defined, this split is described as speciation. Microbiologists often assume that these processes are more complicated in archaea and bacteria because species or ecotype boundaries are distorted due to genes transferred between divergent organisms [2], and very different mechanisms that can contribute to the cohesion of groups of organisms [3]. However, gene flow between incipient species is not restricted to microorganisms. For example, in Darwin's finches, frequent introgression between species living on the same island can make the genomes of those species more similar to each other than genomes of finches belonging to the same species living on different islands [4]. Only the genes that determine the ecological adaptation, e.g., regulate beak development [5], reflect the separation into different sympatric species. In bacteria genes that adapt an organism to a niche can become the vanguard to lineage splitting, and the crystallization point within a genome to decrease homologous recombination between divergent lineages [6]. Motivating our study was the hypothesis that the genes important for interaction with the host, e.g., those encoding the T3SS and in particular the effector proteins, which are translocated across the eukaryotic cell membrane and into the host cell cytoplasm [7], might be driving divergence, similar to the genes determining beak morphology in Darwin's finches. Under this hypothesis we expect that the T3SS system genes should more closely reflect ecological niche, whereas housekeeping genes continue to be exchanged between organisms adapted to different niches. A group of related organisms that inhabits a variety of niches would be ideal to test the importance of the T3SS in driving diversity.

*Aeromonas veronii* is ubiquitous in fresh water and is found in association with a variety of vertebrates and invertebrates with both beneficial and pathogenic outcomes [8], [9], [10], [11], [12]. This species has been divided into two biovars, *Aermonas veronii* bv. veronii and *Aeromonas veronii* bv. sobria, with the latter being considered more virulent [10]. The taxonomic position of a closely related species, *Aeromonas allosaccharophila*, has been debated [13], [14], [15], [16], with some investigators considering them to be synonymous with *A. veronii* [14] and others as separate species [17], [18]. For simplicity, we refer to these related organisms as members of the *Aeromonas veronii* Group (AVG). Of the *Aeromonas* species, *A. veronii* has exhibited the greatest range in virulence as measured in LD_50_ values in a mouse septicemia model [19]. This species has also been reported to cause wound infections, diarrhea and life threatening septicemia in humans [9]. In fish, *A. veronii* has been reported to be a pathogen and also a digestive-tract symbiont of Zebra fish [9], [20]. In addition, *A. veronii* is the digestive-tract symbiont of the medicinal leech and it can also cause wound infections in patients receiving leech therapy if the tissue is poorly vascularized [21], [22]. The wide range of habitats exploited by *A. veronii* strains indicates that this species is a generalist.

While most studies have focused on the role of T3SS in virulence, T3SS are also critical for some beneficial bacteria to colonize their host [23]. For the *A. veronii* strain HM21, a functional T3SS is required for symbiotic colonization of the medicinal leech and virulence in mice [24]. In *Aeromonas salmonicida*, the T3SS and the effector AexT are critical for virulence in fish [25], [26], [27]. In *Aeromonas hydrophila*, the T3SS system is important for virulence in mice, however this species harbors AexU as an effector, not AexT [28], [29], [30], raising the possibility that AexT and AexU maybe involved in virulence with different animals. All of the 20 *A. veronii* strains tested [31], possess both AexU and AexT, which were found together at a locus that was separate from the locus encoding the T3SS [31]. The high prevalence of the T3SS could contribute to the AVG's ability to interact with a wide range of hosts. Interestingly, microbes with an extensive host range often pose the greatest risk to humans and are more likely to be categorized as an emerging and reemerging pathogen [32].

The availability of symbiosis and virulence models, the medicinal leech, *Hirudo verbana* [33] and pathogenic associations using *Galleria mellonella* larvae [34], respectively, combined with strains collected from different sources makes the AVG an ideal model to evaluate whether the lifestyle of the bacteria is reflected in the evolutionary history of the organisms. This history is usually reconstructed using multilocus sequence typing (MLST). Three housekeeping genes, *dnaJ*, *gyrB* and *recA*, and *chiA*, a chitinase were sequenced to reconstruct the phylogeny compared it to the phylogeny constructed from three structural T3SS genes *ascV*, *ascFG*, and the two known effectors, *aexT*, and *aexU* from 20 isolates of the AVG that we obtained in a previous study [31]. The genes involved with host interaction might drive niche adaptation and spearhead strain divergence [35] and thus may reveal niche specificity first.

## Results

### Host specialization of the *A. veronii* group

The AVG strains used in our study were obtained from patients, veterinary samples and medicinal leeches (Table 1). Our initial goal was to evaluate whether eleven of these strains had a similar capacity to associate with host animals or if there was specialization. We evaluated the capacity of these isolates to establish beneficial and pathogenic associations by assessing their ability to colonize the digestive tract of the medicinal leech and kill *G. mellonella*, respectively.

**Table 1:** *Aeromonas veronii* group isolates used in this study.

Species	Strain	Source of Isolation
*A. veronii* bv. veronii	ATCC 35624^T^	sputum of drowning victim
	AER397	blood
	AMC34	human feces
	AMC35	under eye wound
*A. veronii* bv. sobria	LMG13695	feces
	AER28	feces
	AER39	blood
	AMC22	feces
	AMC23	finger wound
	AMC24	feces
	AMC25	duck
	AMC26	foot wound
	Hv221	leech (*Hirudo verbana*)
	Hv231	leech (*H. verbana*)
	Hv241	leech (*H. verbana*)
	HM21	leech (*H. verbana*)
	Hv648	leech (*H. verbana*)
	Ho635	leech (*Hirudo orientalis*)
	Ho636	leech (*H. orientalis*)
*A. allosaccharophila*	LMG140549^T^	eel

#### (i) Symbiotic competence

**Figure 1:**
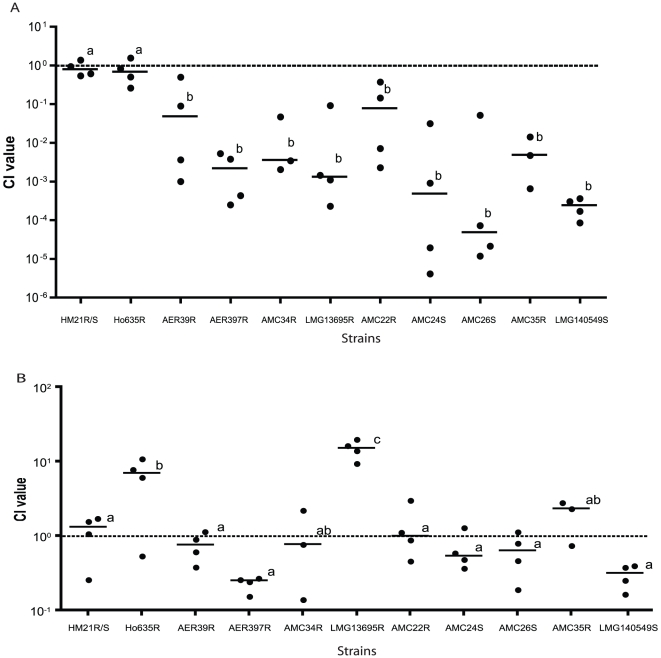
Symbiotic competence of AVG isolates and ability to grow in blood. (A). The ability of AVG isolates to colonize the leech. Spontantous antibiotic resistant isolates were coinoculated with the competitor strain (HM21R or HM21S) in a 1∶1 ratio. The antibiotic resistance of the strain is indicated by “R” for rifampin resistant and “S” for streptomycin resistant. The CI [(test_output_/competitor_out_)/(test_input_/competitor_input_)] was calculated. Each point represents the CI value from a single competition assay. A CI of 1 (dashed line) indicates that the test strain and competitor strain colonize to equal levels. A CI below 1 indicates that the test strain is outcompeted and has a colonization defect. The CI for each animal is shown. Horizontal lines represent median CI values. Strains with common letters are not statistically different from one another as determined by one-way ANOVA with Tukey's post-hoc test, *P*<0.05. (B). Blood was coinoculated with an AVG isolate and the competitor strain (HM21R or HM21S) in a 1∶1 ratio. The CI was calculated. A CI of 1 (dashed line) indicates that the test strain and competitor strain proliferate to equal levels. A CI below 1 indicates that the test strain is outcompeted and has a defect in its ability to grow in blood. Horizontal lines represent median CI values. Strains with common letters are not statistically different from one another as determined by one-way ANOVA with Tukey's post-hoc test, *P*<0.05.

Symbiotic competence was assessed in a standard competition assay [24]. A spontaneous antibiotic resistant derivative of the AVG strain and a leech isolate with a different antibiotic resistance marker were added at an equal concentration to a blood meal that was fed to the leech, *H. verbana*. The animals were sacrificed 42 h after feeding and the relative numbers of the strains determined by plating on antibiotic containing plates. A competitive index (CI) was calculated (Test Strain_output_/Competitor_output_/Test Strain_input_/Competitor_input_). The different antibiotic resistance markers did not change symbiotic competence as shown when HM21R, a spontaneous rifampin resistant mutant derived from HM21, and HM21S, a spontaneous streptomycin resistant mutant derived from HM21, were competed against each other and a competitive index of 0.86 was obtained. This value was not significantly different from 1 indicating that neither spontaneous mutation had an effect on the ability to colonize the leech. In a previous study, Ho635, isolated from *Hirudo orientalis* (Table 1, Figure 1A), was shown to possess a CI of 0.79, which indicated that it colonized to comparable levels as the competitor strain [36]. All of the remaining isolates, which were not isolated from leeches, had a statistically significant reduced ability to colonize the leech (Figure 1A). The CI values for all of the non-leech isolates exhibited a large range in the severity of their colonization defect (Figure 1A). Two strains, AER39 and AMC22, possessed slight but significant symbiosis defects (<10-fold), while LMG140549^T^ had a>4,000-fold defect (Figure 1A). These data indicate that isolates cultured from the leech out-compete strains from other sources when colonizing their native host and suggest host specialization.

#### (ii) Growth in blood

It is possible that the growth defect inside the leech was due to an intrinsic property of the leech crop or a general inability to proliferate in blood. The CI values for the 11 strains were determined in heat-inactivated blood (Figure 1B). HM21R produced a mean CI value of 1.16 when competed against HM21S, indicating the spontaneous antibiotic resistance mutations do not have an adverse effect on either strain's ability to proliferate in heat-inactivated blood (Figure 1B). The CI values for eight of the strains were comparable to the CI for HM21 (Figure 1B). Two strains, Ho635 and LMG13695, had statistically significantly higher CI values than HM21, 6.73 and 15, respectively, which indicated that these two strains grew better in blood than the leech isolate (Figure 1B). Therefore, the colonization defect in the leech is not due to a reduced ability to grow in blood but due to a property intrinsic to the leech digestive-tract habitat.

#### (iii) Virulence

**Figure 2:**
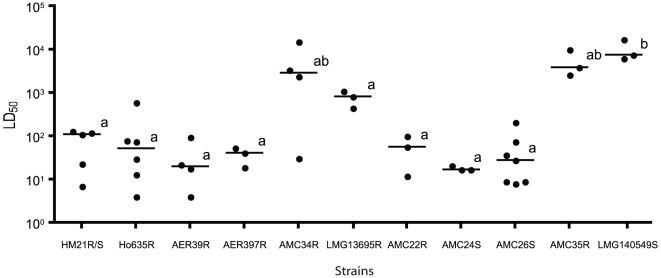
Virulence of AVG isolates in the *Galleria mellonella* infection model. The LD_50_ was calculated for each isolate 24 h after *G. mellonella* larvae were inoculated using the Reed-Muench method [59]. Horizontal lines represent median LD_50_ values. Strains with common letters are not statistically different from one another as determined by one-way ANOVA with Tukey's post-hoc test, *P*<0.05.

The greater wax moth caterpillar, *G. mellonella*, has previously been used as a model to study the virulence of a variety of bacterial, viral, and fungal pathogens [37]. For example, in two instances bacterial virulence has been correlated between *G. mellonella* and a mammalian model [38], [39], which demonstrates the usefulness of this model system in assessing bacterial virulence. *G. mellonella* possesses hemocytes, macrophage-like cells of invertebrates, and antimicrobial peptides, which for our study, made an ideal assay to examine the ability of different isolates to evade the innate immune response and cause disease in an animal model. The LD_50_ was determined for eleven strains from the AVG, using *G. mellonella* as the host animal, in order to assay for differences in virulence between the strains. While there was a large range in virulence with AMC24 being the most virulent strain, with a mean LD_50_ of 18 CFU and LMG140549^T^ being the least virulent with an LD_50_ of >10,000 CFU, all but LMG140549^T^ were statistically indistinguishable from each other (Figure 2). Two strains with mean LD_50_ values>5,000 CFU also grouped with LMG140549^T^ (Figure 2). Most strains had a mean LD_50_ between 18 and 134 CFU suggesting that overall the strains examined have the potential to be virulent and overpower the innate immune system (Figure 2).

### Sequence analysis

We wanted to determine the phylogenetic relationship of these strains by sequencing four genes, *gyrB*, *recA*, *dnaJ* and *chiA* and compare that phylogeny to one obtained from genes associated with the T3SS: *ascFG*, *ascV*, *aexT* and *aexU* (Table S1). Initial maximum-likelihood and Bayesian trees of each gene family provided low bootstrap support (not shown) and thus we employed different strategies to reveal the evolutionary relationship of these strains.

#### (i) Lack of agreement of phylogenetic signal

Comparisons of the inferred maximum-likelihood topologies against a concatenation of all gene alignments using the Approximately Unbiased test rejected the null hypothesis of congruent phylogenetic signals for all families (p≤0.01) implying different evolutionary histories among this group of genes. A tree topology estimated as having the maximum likelihood for a sequence alignment may be contained within a set of topologies with very similar, high likelihoods but may perform more poorly than others in that set when tested with a different sequence alignment. For this reason additional permutations (see Methods) were included in the testing to decrease the chance of being misled by the result from the most likely topology. None of the permutations for non-concatenated gene families passed the AU test (Table S2).

**Figure 3:**
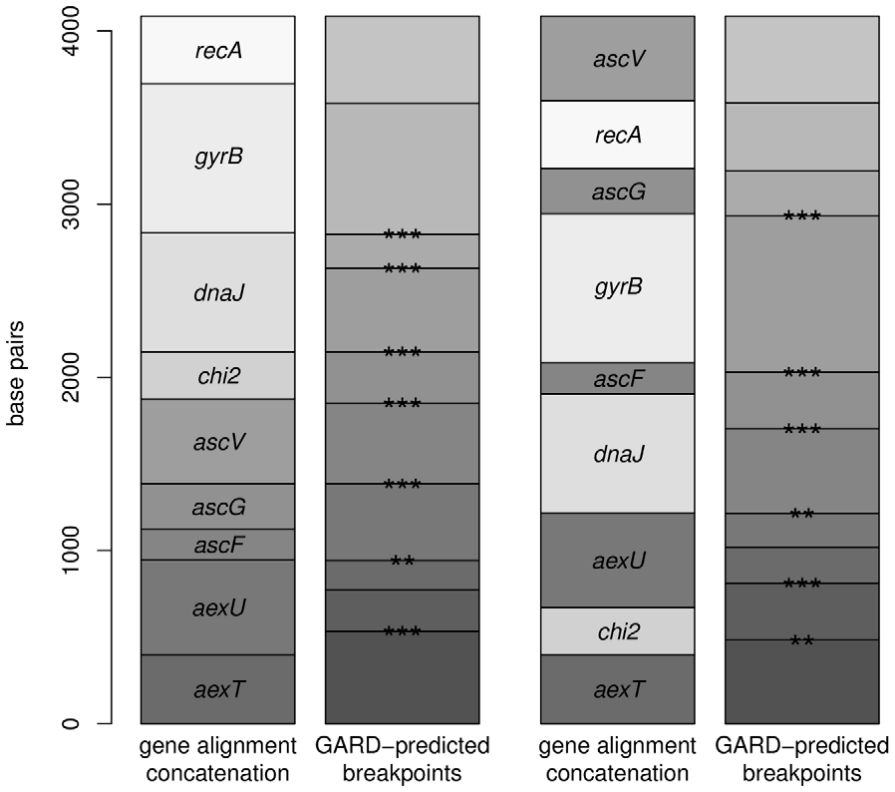
Gene maps of two differently ordered concatenations of the nine gene families with predicted breakpoints. GARD inferred recombination breakpoints delimit regions with distinct phylogenies. Significance of breakpoints according to KH test: ***, *Ρ*<0.01; **, *Ρ*<0.05; * *Ρ*<0.1; no asterisk, not significant.

One explanation for individual gene families failing the AU test is that individual genes may not share a common evolutionary history but evolved as fragments. A Genetic Algorithm for Recombination Detection (GARD) [40] analysis applied to two differently ordered concatenations of all nine gene families predicted more fragments than gene families in both instances (Figure 3). This analysis compares the goodness of fit of phylogenies inferred from alignment fragments under the maximum likelihood framework using the corrected Akaike Information Criterion. With the exception of *recA* and *aexT*, significant breakpoints were predicted for all gene boundaries in at least one concatenation configuration providing further support for differing evolutionary histories among this group of genes (Figure 3). Interestingly, internal breakpoints were predicted for both concatenations within both *dnaJ* and *aexU*. A breakpoint was not predicted between *ascF* and *ascG* in the first concatenation configuration indicative of similar evolutionary histories (Figure 3). These two gene sequences were PCR amplified on the same fragment.

#### (ii) Recombination and break-point detection

The aligned sequences were investigated for evidence of recombination because the maximum-likelihood inferred phylogenies for each gene family showed low bootstrap support for most clades. One explanation for low bootstrap support is that different gene families yield incompatible phylogenies. Two tests for recombination employing different approaches were applied (Table 2) because different approaches have different advantages and drawbacks [41], [42], [43] The Pairwise Homoplasy Index (PHI) [44] depends on pairwise genealogical correlation of adjacent sites within a window and GARD analysis with KH test assesses the topological congruity of the inferred phylogeny. Neither test detected recombination in the *chiA* or *aexT* sequences. Both tests detected recombination in the *ascFG*, *aexU*, *ascV*, *gyrB* and *dnaJ* sequences. The results for both tests were in agreement for all sequences tested except for *recA* and evidence for recombination was found in both housekeeping and T3SS associated gene sequences.. The GARD analysis predicted a recombination break point at 126 bp of *ascFG*, not between *ascF* and *ascG*, in *ascV* at 226 bp, *dnaJ* at 483 bp and *recA* at 177 bp. Two break points were inferred for *aexU* at 153 bp and 349 bp, and *gyrB* at 249 bp and 528 bp, all at a significance of p<0.01 for the KH test.

**Table 2:** Tests for recombination applied to each gene family.

Gene	PHI^a^ p-value	No. of GARD fragments^b^	KH-test^c^ p-value	Both tests support recombination?
*aexT*	0.326	1	N/A	No
*aexU* *	<0.001	3	<0.01	Yes
*ascFG* *	<0.001	2	<0.01	Yes
*ascV* *	0.006	2	<0.01	Yes
*chiA*	0.168	1	N/A	No
*dnaJ* *	<0.001	2	<0.01	Yes
*gyrB* *	0.025	3	<0.01	Yes
*recA*	0.425	2	<0.01	No

*These alignments returned significant results (p<0.05) for recombination in both tests and were analyzed as separate GARD-predicted fragments in quartet decomposition to avoid conflicting phylogenetic signals.

^*a*^PHI, Pairwise Homoplasy Index. P-values are for a null hypothesis of no recombination.

^*b*^GARD, Genetic Algorithm for Recombination Detection.

^*c*^KH, Kishino-Hasegawa test performed on phylogenies inferred from alignments between break points predicted by GARD. P-values are for a null hypothesis of congruent phylogenies *i.e.*, no recombination. N/A – not applicable, where no recombination break points were detected.

#### (iii) Evolutionary history by Quartet Decomposition

The evolutionary history and the prevalence of recombination among specific strains was determined by performing a quartet decomposition analysis on the aligned sequences with *ascFG*, *aexU*, *ascV*, *dnaJ* and *gyrB* separated at predicted recombination breakpoints (see Table S1 for a summary of sequences analyzed) Separate analyses were performed including only housekeeping gene sequences (*chiA*, *gyrB*, *dnaJ*, and *recA*) and only T3SS gene sequences (*aexT*, *aexU*, *ascFG*, *ascV*). Four-tipped, unrooted, bifurcating trees (quartets) describe non-trivial split relationships and are the smallest units of phylogenetic information. Embedded quartets allow better resolution of sequence relationships than bipartitions in a full topology with respect to bootstrap support [45], [73]. The quartets embedded in each of the 100 bootstrap replicate trees for each aligned fragment were extracted. The most common relationships of each quartet across all trees were combined by a supertree-like method (QNet) [45] as splits networks depicting the consensus signal to reveal any departure from a common tree-like history for these sets of genes.

**Figure 4:**
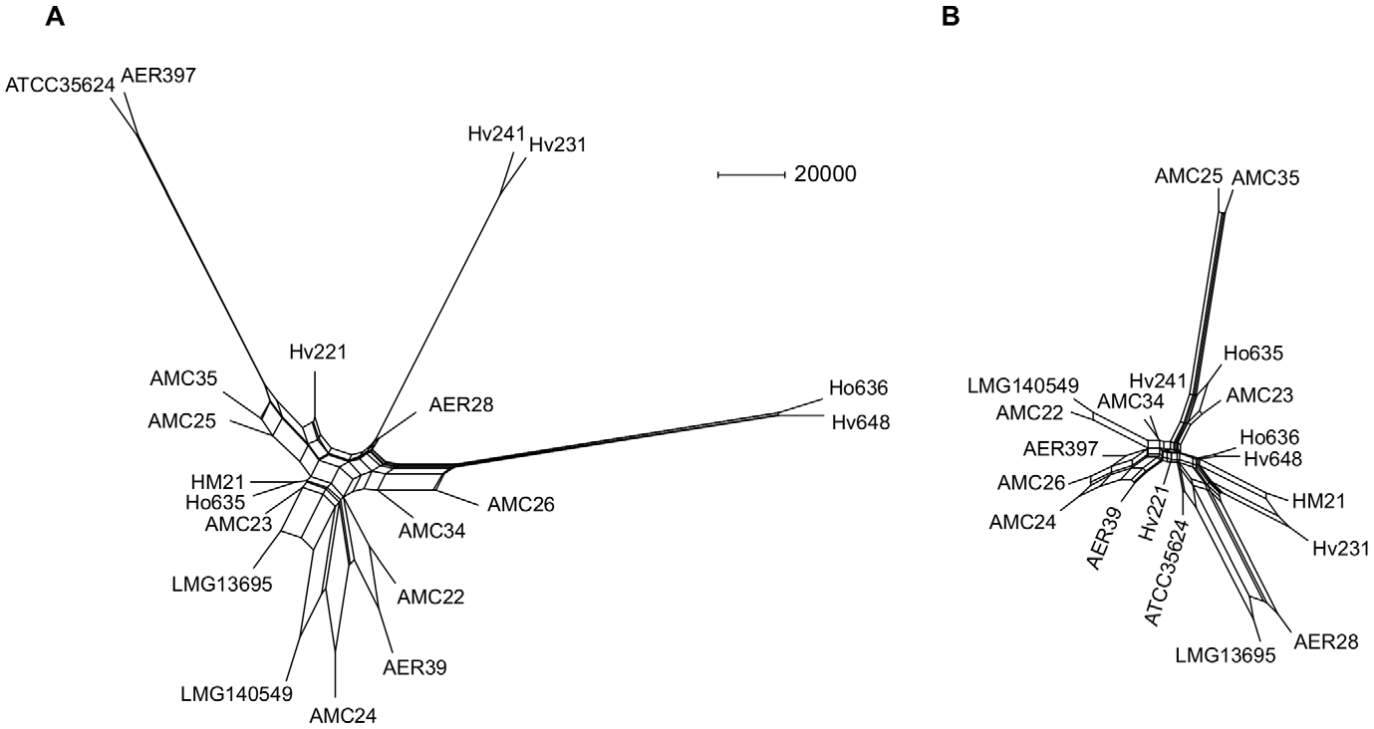
Split networks generated using QNet with embedded quartets compatible with the plurality consensus signal. These evolutionary relationships were inferred from maximum likelihood trees of 100 bootstrap replicates, weighted by frequency, of (A) house-keeping gene sequences (*chiA*, *gyrB*, *dnaJ*, and *recA*) and (B) T3SS gene sequences (*aexT*, *aexU*, *ascFG*, *ascV*). Alignments were divided at predicted recombination breakpoints determined by distinct evolutionary histories. Edge lengths are scaled to the number of embedded quartets in agreement with that split and split widths represent a departure from a tree-like signal. In the case of *Aeromonas veronii* group strains splits with width are likely to be indicative of recombination across that split.

Figure 4 shows splits networks of each set of fragments clustered according to the inferred evolutionary history of the tested *A. veronii* group strains. The circular ordering of terminal nodes is dictated by evolutionary distance and internal nodes do not represent ancestral states. The lengths of the edges of the networks are scaled to the number of bootstrap replicate embedded quartets supporting that split. The biological interpretation of conflicting quartets causing a non-treelike signal in this scenario is that recombination has occurred. The graphical representation of recombination between taxa are parallel edges leading to strains instead of single edges in the absence of recombination. The width of parallel edges is proportional the amount of inferred recombination but does not specify donor-recipient relationships.

In the analysis of all gene fragments, AMC25 and AMC35, Ho636 and Hv648, and ATCC35624^T^ and AER397 each form well-resolved groups with few conflicting quartets. HM21, Hv231 and Hv241 and AER28 and LMG13695 form two moderately well resolved groups with some evidence of recombination (signal conflict). Ho635, AMC23, AER39, AMC22, AMC24, AMC26, and LMG140549^T^ form a less clearly defined group with the last 5 members showing greater evidence of recombination.

LMG13695 and AER28, and AMC25 and AMC35 form well resolved pairs according to the T3SS sequences but not the housekeeping sequences. ATCC35624^T^ and AER397 and Hv241 and Hv231 are well resolved by the housekeeping sequences but not the T3SS sequences. Ho636 and Hv648 are well resolved by the housekeeping genes and weakly resolved by the T3SS genes. Overall there is little congruity between the evolutionary relationships inferred from the two sets of sequences. Within the housekeeping genes there is evidence of recombination (phylogenetic signal conflict) among all strains except ATCC35624^T^, AER397, Hv241 and Hv231. Ho636 and Hv648 show evidence for a relatively small degree of recombination. Within the T3SS genes there is evidence for recombination among all strains except AMC34, Hv241, Ho636 and Hv648. Figure S1 panel A shows an embedded quartet splits network of the combined signal. It indicates some groups are well resolved with little between-group gene exchange since the last common ancestor of the sampled strains, *e.g.*, Ho636 and Hv648; AMC25 and AMC35. Other groups have undergone recombination, *e.g.*, AMC26, AMC24 and AER39. Further characterization of the sequences with respect to information content and agreement of signal is provided in Analysis S1, Table S3, Table S4 and Figure S2.

### Comparison of source, phenotypic data and phylogenies

Neither the source of the strains, nor their ability to colonize the leech, grow in blood, or cause disease in wax moth larvae correlated in a consistent manner with the phylogenies we obtained. This was explicitly tested by plotting the proportion of embedded quartets in agreement, against the proportion in disagreement with the groups inferred from the phenotypic data (Figure S2; see methods for group demarcation; [46]). For leech colonization the groupings were split by (Ho635, Hm391 | AMC34, AMC35, AMC22, AER39 | AMC24, AMC26, LMG13695, LMG140549^T^, AER397), for growth on blood by (LMG13695, Ho635 | LMG140549^T^, AER397 | HM21, AMC22, AMC26, AER39, AMC24, AMC34), and for LD_50_ in *G. mellonella* by (LMG140549^T^, LMG13695, AMC34, AMC35 | Ho635, AER397, AER39, AMC22, AMC23, AMC24, AMC25, AMC26). Strong disagreement for the embedded quartets of a particular fragment with a particular phenotypic group near the top left of the plot (y≈1) and agreement near the bottom right (x≈1). The combined agreement plus disagreement score cannot exceed 1. Lack of phylogenetic information or groupings for which some quartets are not informative (non-trivial splits) will result in points nearer the origin. The points are distributed along a line representing equal proportions of disagreement with agreement (for each quartet of taxa there is one topology that can agree with a specific partitioning but two that can disagree hence the slope of 2) and are near the origin (x and y<0.18). There is a lack of signal in either direction for each set of phenotypic groups inferred rejecting the hypothesis that the evolution of host interaction is linked to the gene families tested (Figure S3).

**Figure 5:**
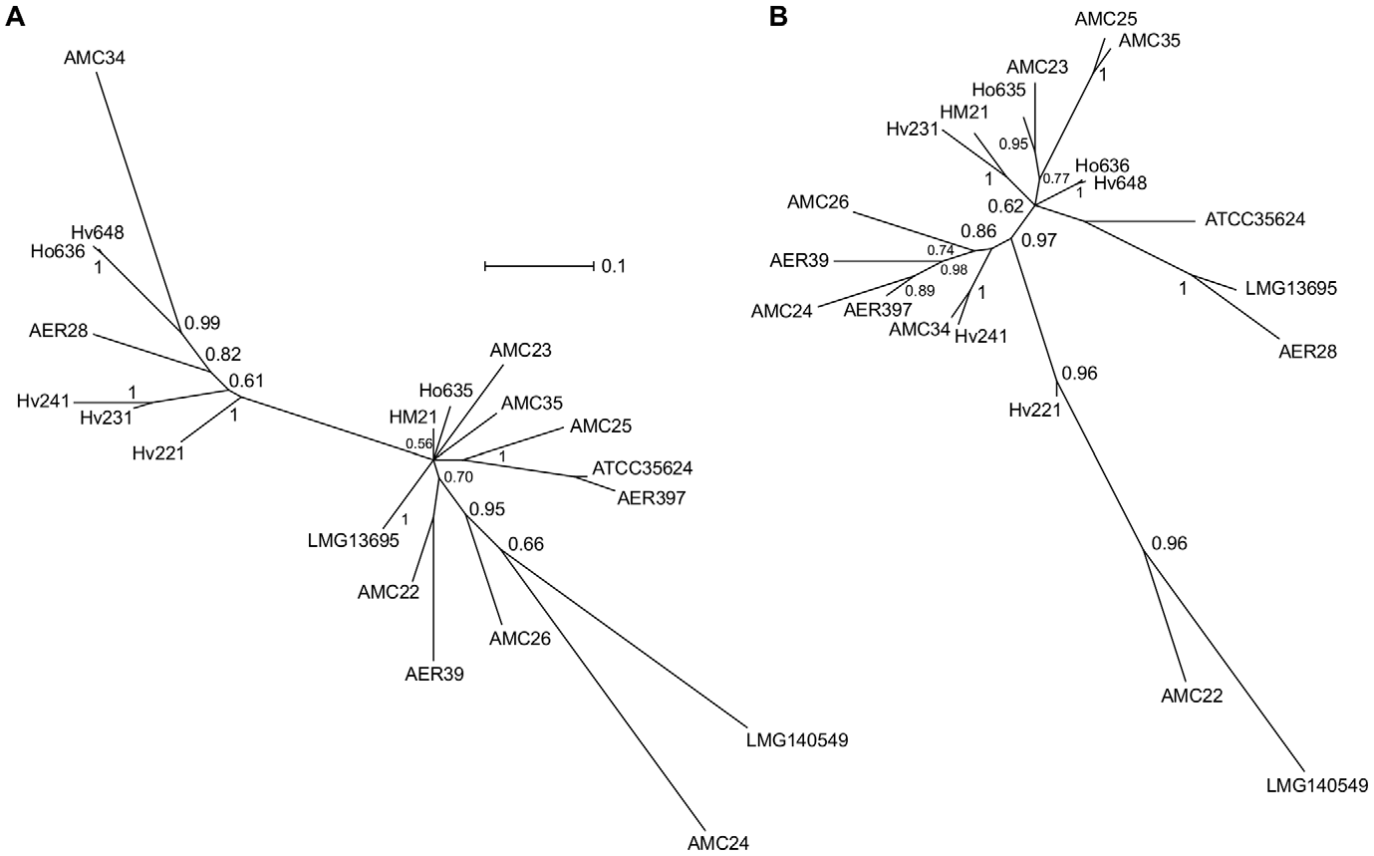
Bayesian trees inferred from two concatenations of gene family alignments. **A**. housekeeping gene sequences (*chiA*, *gyrB*, *dnaJ*, and *recA*) and **B**. type III secretion system gene sequences (*aexT*, *aexU*, *ascFG*, *ascV*). Numbers represent the posterior probability for that split, edge lengths are scaled to average substitutions per site in the posterior distribution.

This lack of correlation occurred despite clear adaptation to the leech environment by isolates obtained from leeches. Experiments to investigate the traits related to host association included growth in blood for which Ho635 and LMG13695 were significantly different from each other and to all other strains, and LD_50_ (*G. mellonella*) for which LMG140549^T^ was distinct from all strains except AMC34 and AMC35. Neither Ho635 nor LMG13695 are distinctly resolved by the sequence data. Figure 5C indicates LMG140549^T^ to have more divergent T3SS sequences.

## Discussion

Previously it has been shown that DNA-DNA hybridization and 16S rRNA gene sequencing analysis have produced inconsistent results in differentiating *Aeromonas* strains at the species level [16], [47]. The 16S rRNA gene is not considered to be an ideal marker to use for taxonomic analysis at the species level, especially within the genus *Aeromonas* due to the presence of several copies and intragenomic heterogeneity, which is suggestive of horizontal gene transfer [16], [47], [48]. Other studies have attempted to reconstruct the phylogeny of the genus *Aeromonas* using individual genes (*cpn60*, *dnaJ*, *gryA gyrB*, *mdh*, *recA*, *rpoB* or *rpoD*) or combined several of these genes but did not perform rigorous tests to evaluate if the individual phylogenies were consistent [16], [17], [49], [50], [51], [52], [53].

This study focused on a subgroup of *Aeromonas*, AVG, where isolates had been obtained from different sources and showed specialization in the ability to colonize the leech. Eight different loci were utilized to determine whether the phylogeny of these strains would reflect host specialization that were revealed using phenotypic tests [3]. Despite subsets of strains performing differently in particular phenotypic tests, which is reflective of these strains being specialized for a particular niche, the sequence data were not consistent with these strains clustering together. Instead, the quartet decomposition analysis indicates a complex recent evolutionary history for these strains with a high frequency of horizontal gene transfer between several strains but not among all strains. After excluding potentially ambiguous quartets, the remaining quartets that provided a signal were a minority of the initial set, a substantial proportion of them disagreed with the plurality consensus phylogenetic signal, and those that did agree combined to form a non-tree-like signal. Combined with the evidence of recombination found in several of the PCR fragment alignments it is likely that at least some of these strains share a history of horizontal gene transfer with chromosomal integration by homologous recombination.

One strain included in our study, LMG140549^T^, was identified as *A. allosaccharophila*. Whether *A. allosaccharophila* is a distinct species has been debated for over a decade [13], [14], [15], [16], [17], [18]. The quartet decomposition analysis indicated that for housekeeping genes and the T3SS this strain readily shared DNA with *A. veronii* strains consistent with it being synonymous with *A. veronii* as some research groups have suggested [16], [54]. It is of interest to note, however, that the T3SS associated genes alone suggest this strain to be distinct from most AVG strains (Figure 4 and 5). A comparison of rooted Bayesian phylogenetic trees inferred from *aexU* recombinant fragments with that of a concatenation of housekeeping genes further illustrates this (Figure S1). Additional DNA–DNA hybridization experiments with a greater diversity of AVG strains might help to resolve the taxonomic status; however, if the divergence between *A. allosaccharophila* and the remainder of the AVG group represents a speciation in progress, with as yet incomplete barriers to gene flow and homologous recombination, then a detailed comparison of complete genome sequences when available, as performed by Retchless and Lawrence [6] for the *Escherichia* - *Salmonella* divergence, might be able to pinpoint the genes that drive the speciation process.

In contrast to the molecular data, both the LD_50_ (*G. mellonella*) and growth in blood data suggest *A. allosacchrophila* LMG140549^T^ as being distinct from the other strains. Its T3SS sequence data also differs but is closest to that of AMC22, which is an *A. veronii* bv. sobria strain that is virulent. Yet, the T3SS from the two strains that were not significantly different from LMG140549^T^ in regard to virulence, AMC34 and AMC35, clustered with other strains. This suggests the lack of a clear-cut association with the lack of virulence.

The split networks inferred from the housekeeping gene sequences and T3SS sequences each indicate three pairs of strains having a high frequency of supporting splits in the embedded quartets of the bootstrap tree replicates. A difference between the two networks is that the well-supported pairs in the latter network are connected with parallel edges to the rest of the network but with single edges in the former. Although there are a similar number of predicted recombination breakpoints throughout the two sequence sets, the predicted recombinant fragments within the T3SS sequences contain a more conflicting phylogenetic signal. Based on this sample, it is possible that recombination between more divergent T3SSs is more strongly selected for than similar recombination between housekeeping genes. To test this hypothesis a comparative analysis of complete genome sequences would be necessary.

The Bayesian tree reconstruction method [55] merges the conflicting signals contained in the data set and forces the non-tree-like signal onto a bifurcating tree model. It fails to indicate non-tree-like signals that in the case of AER28 and LMG13695 may have resulted in an artifactual placement either side of a clade containing Hv241, Hv231 and Hv221 (Figure 5A), compared to the quartet decomposition plurality signal inferred split network (Figure 4A). The quartet decomposition method combined with recombination break-point detection avoids merging conflicting phylogenetic signals, explicitly removes noise to decrease the risk of artifacts and allows plotting the resulting majority signal as a splits network to indicate differences among strains with respect to non-tree-likeness *i.e.*, evidence for recombination. A drawback in using quartets is that trivial splits (those dividing a single sequence from others) are not processed which may have resulted in the placement of AMC34 in the central polytomy of figure 4A but on a relatively long branch in figure 5A and concealed the evolutionary distance between LMG140549^T^ and the other T3SS sequences in figure 4C.

The 20 isolates of the AVG included representatives from *A. veronii* bv. veronii and *A. veronii* bv. sobria. Regardless of the network, there appears to be no signal that lets one infer consistently the biovar of the strains although a small proportion of each genome is used in this analysis. Even though there are phenotypic differences between the isolates, there is not a consistent signal in the multilocus sequence analysis that allows one to differentiate the biovars. This lack of resolution in and between the networks can be explained because our analysis suggests there are different evolutionary histories among the groups of genes, there is evidence for recombination in both housekeeping and T3SS associated genes, and overall the data set is characterized by a low phylogenetic signal. Taken together, *Aeromonas* phylogenetics and in some instances taxonomic classification is not clear, therefore future studies need to be carefully constructed and encompass multiple strategies in order to prevent the reporting of inaccurate data.

Many bacteria have been shown to associate with multiple hosts *e.g.* the opportunistic pathogens *Yersinia pestis* and enterohemorrhagic *Escherichia coli* and have different outcomes even with the same host. These differences are often thought to be due to acquisition of particular virulence factors such as a T3SS, hemolysins or other toxins and with sufficient time one might expect a molecular signal in the genome of the strain, which could be revealed by sequencing housekeeping genes. In our case no such signal could be detected because the apparent high rate of gene flow. This may be feasible for organisms that occupy multiple niches, where strains specialized to particular environments can encounter each other. So even in the case of leech symbionts, which are vertically inherited, high levels of horizontal gene transfer occurred [56]. It will be of interest to sequence genomes of these strains and determine if there are individual genes that determine host range and how large the regions are that transferred between strains.

## Materials and Methods

### Growth conditions and DNA isolation

Strains were grown and DNA was isolated from the 20 AVG isolates as previously described [24], [33].

### PCR amplification of the T3SS and housekeeping genes

The T3SS associated genes, *ascV*, *ascFG*, *aexT* and *aexU* [31] and the housekeeping genes *dnaJ* [16] and *gyrB* [52] were amplified as previously described. The primers chiA_2F 5′-CACCAAGTTYGCCATCGTTGAAG-3′ and chiA_2R 5′-GCCGGGATCTTGTCSACGGT-3′ and recA_2F 5′- GAAGCCATCTCTACCGGTTC-3′ and recA_2R 5′-CCGTTATAGCTGTACCAGGCACC-3′ were used to PCR amplify *chiA* and *recA*, respectively [57]. The PCR reaction mixture contained approximately 100 ng of DNA, 1× PCR buffer, 1.5 mM MgCl_2_, 200 µM of each dNTP, 0.2 µM of each primer, and 1 U of Platinum *Taq* DNA polymerase (Invitrogen, Carlsbad, CA) in a final volume of 50 µl. The amplification conditions for *chiA* were comprised of an initial 2 min denaturation step at 94°C followed by 30 cycles of 30 s at 94°C, 30 s at 62°C and 30 s at 72°C. The amplification conditions for *recA* comprised of an initial 2 min denaturation step at 94°C, followed by 30 cycles of 30 s at 94°C, 30 s at 58°C and 1 min at 72°C.

### DNA sequencing

DNA was sequenced as previously described [24]. Contiguous DNA sequences were assembled using ContigExpress and analyzed using VectorNTI 7. The DNA sequences for *dnaJ*, *recA*, *gyrB*, *chiA*, *ascV*, and *ascFG*, obtained in this study were deposited in GenBank (accession numbers HM584488–HM584607).

### Competition assays

Spontaneous rifampin or streptomycin-resistant mutants were obtained as described previously [22], [33]. In order to verify none of the spontaneous resistance mutants possessed a general growth defect, the growth rate was determined at 30°C in a gyrator shaker in LB [24], [58]. The competition assay used in this study compares the colonization ability of a test strain against a competitor strain, HM21S or HM21R, which were derived from HM21, an isolate from the digestive tract of *Hirudo verbana* [22]. The test strains consisted of the spontaneous resistance mutants derived from 11 AVG strains. The conditions of the competition assay were identical to those for the assay described previously [33]. At least three animals were examined 42 h post feeding. The limit of detection was 10 CFU/ml.

### Growth in Heat-Inactivated Blood

The growth yield in heat-inactivated blood was assessed by removing an aliquot of the inoculated blood from the competition assay and incubating it at room temperature (23°C) for 42 h. An aliquot was then removed, serially diluted, and plated as previously described for the competition assay [11].

### *Galleria mellonella* virulence assay

Strains were grown overnight at 30°C in LB and subcultured the following morning. 10^8^ cells from mid-log phase were spun down and resuspended in 1 ml of 10 mM MgSO_4_ [34]. Five 10-fold serial dilutions were performed and the inoculum determined by plating on LB agar in duplicate and incubating overnight at 30°C. *G. mellonella* larvae were placed in a Petri dish and kept on ice in order to keep larvae stationary during injection. A 10 µl Hamilton syringe (model number 701RN) was used to inject 5 µl from each dilution into the left hindmost proleg of the larvae. In between inoculations the syringes were sterilized with 70% EtOH and rinsed with 10 mM MgSO_4_. Six larvae were used per dilution. Larvae inoculated with 10 mM MgSO_4_ served as a control. After inoculation, larvae were kept at room temperature for 24 h. The LD_50_ was calculated 24 h after inoculation using the Reed-Muench method [59].

### Sequence analysis

Nucleotide sequences were translated to protein sequences and aligned first with ClustalW [60] using the default settings then with the refine option in MUSCLE [61]. The nucleotide sequences were aligned to the protein alignments using Tranalign in the EMBOSS package [62]. Subsequent analyses were performed on the nucleotide sequences. Nucleotide substitution models were selected for each alignment using the ‘phymltest’ function in the Analysis of Phylogeny and Evolution (APE) [43], [63] package for the R statistical environment [64].

### Shared phylogenetic signal

The Approximately Unbiased (AU) test, as implemented in the ‘scaleboot’ package for R [65], was applied between sets of similar tree topologies from an alignment and the concatenated alignment. If all alignments passed, a shared phylogenetic signal was inferred. Three sets of similar topologies were generated per alignment and consisted of the maximum-likelihood phylogenetic tree inferred using PhyML [66] and 5 additional permutations by one or two random nearest neighbor interchanges (NNIs) or a random subtree prune and regraft (SPR) respectively. Permutations were achieved using the ‘rNNI’ and ‘rSPR’ functions in the ‘phangorn’ package for R (http://CRAN.R-project.org/package=phangorn). Site-wise log-likelihood calculations were performed using TREE-PUZZLE [67]. A Genetic Algorithm for Recombination Detection (GARD) [40] implemented in HyPhy [68] via the DataMonkey web interface [69] was applied to a concatenation of all families (restricted to the 18 common sequences) as a further test for shared phylogenetic signal, estimating substitution frequencies and a proportion of invariable sites from the data with 4 rate categories in a beta-gamma distribution. This implementation of GARD includes the Kishino-Hasegawa (KH) test for tree congruence [70].

### Recombination

Evidence for recombination within each gene family alignment were tested for with the Pairwise Homoplasy Index (PHI) [44] and GARD [40] using nucleotide substitution models selected above and estimating substitution frequencies and a proportion of invariable sites from the data with 4 rate categories in a gamma distribution. The latter was also used to locate recombination breakpoints and *ascF* and *ascG* were treated as a single sequence.

### Evolutionary relationships

A Quartet Decomposition supertree [46], [71], [72] and a total evidence approach [55] were used. Quartet Decomposition was performed on 100 topologies per gene family inferred by maximum likelihood from 100 bootstrap samples of each alignment using phyML [66] using the nucleotide substitution models selected above and estimating substitution frequencies and a proportion of invariable sites from the data with 4 rate categories in a gamma distribution where required by the model. Scripts for this task were written in R using functions from the APE package [63] with further processing using Perl. Considering average values across bootstrap replicates, quartets were excluded with less than 3 substitutions along the internal edge to avoid ambiguous quartet topologies and with an external edge length (on the original topology) more than 10 times the length of the internal edge to avoid long branch attraction artifacts [73]. The plurality topology for each quartet was determined by the total bootstrap support score for each possible topology across gene families scaled to the number of characters (nucleotides). Additionally, quartets that were resolved in less than 30% of gene families were excluded [71].

Quartet decomposition analysis was performed with the 12 predicted recombinant gene fragments, fragments from housekeeping genes and fragments from T3SS genes each separately. The frequency of plurality quartet topologies in the bootstrap replicates across all families (‘bootstrap scores’), scaled to the number of characters (nucleotides) were used as weights for plotting a splits network using QNet [45]. ‘Contribution’ scores for each sequence fragment were calculated as described previously (described there as ‘agreement’ scores) [46], [72]. ‘Agreement’ scores were calculated similarly except normalization was achieved by dividing by the maximum possible score after exclusion of ambiguous quartets (as described above) thus distinguishing between disagreement and poor resolution. ‘Information content’ scores for each family were calculated as the sum of highest bootstrap score of the three topologies for each quartet divided by the maximum possible bootstrap score for that family.

Total evidence based evolutionary clustering was inferred from the concatenated alignment of the three housekeeping genes and chitinase (*dnaJ*, *gyrB*, *recA* and *chiA*), the four T3SS loci and a concatenation of all genes using MrBayes 3.1 [74]. Each codon position and each gene family were allocated to unlinked substitution rate data partitions all allowing 6 substitution types, a proportion of invariable sites, a gamma distribution of variable site rates with 4 rate categories and a uniform prior for the topology and was run for 10,000,000 generations with a sample frequency of 10,000. The first 400 samples were discarded as ‘burn in’. The phylogenetic relationship between *A. allosaccharophila* (strain LMG140549^T^) and members of AVG was investigated in the same way for the concatenation of housekeeping genes and the chitinase gene and for each of the inferred recombinant fragments of *aexU* using *A. hydrophila* as an outgroup to root the trees. Adequate effective sample sizes and MCMC chain convergence were checked using Tracer 1.4.

### Agreement of phenotype with genotype

Strains were partitioned into groups according to beneficial symbiosis and virulence assays. For each assay type strains were ranked according to their median response, and possible partitions of strains into groups were tested for significance using the Mann-Whitney U-test. All of the chosen partitions were significant with *P*<0.0015. Embedded quartets scatter plot analyses were then performed to test the agreement of phenotype groupings with genotype groupings as described previously [46] except a bootstrap threshold was not applied (all quartet scores were included) and the normalization was all quartets possible for each gene family multiplied by the number of bootstrap replicates.

## Supporting Information

Analysis S1 **Identification of Informative Sites.**
(DOCX)

Table S1 **Summary of analyzed sequences.**
(DOC)

Table S2 **Approximately Unbiased test.**
(DOC)

Table S3 **Agreement and information content scores.**
(DOCX)

Table S4 **Contribution and information content scores.**
(DOCX)

Figure S1 **Split networks and Bayesian trees inferred from concatenations gene family alignments.** **A**. These evolutionary relationships were inferred from maximum likelihood trees of 100 bootstrap replicates, weighted by frequency, of *chiA*, *gyrB*, *dnaJ*, *recA*, *aexT*, *aexU*, *ascFG*, and *ascV*. Alignments were divided at predicted recombination breakpoints determined by distinct evolutionary histories. Edge lengths are scaled to the number of embedded quartets in agreement with that split and split widths represent a departure from a tree-like signal. In the case of *Aeromonas veronii* group strains splits with width are likely to be indicative of recombination across that split. **B**. From the same concatenated sequences Bayesian trees were inferred. Numbers represent the posterior probability for that split, edge lengths are scaled to average substitutions per site in the posterior distribution.
(PDF)

Figure S2 **Bayesian trees inferred from housekeeping genes and each** ***aexU*** **recombinant fragment.** (A). Tree inferred from concatenation of housekeeping gene sequences (*chiA*, *gyrB*, *dnaJ*, and *recA*); (B, C and D) trees inferred from each of the three inferred recombinant fragments of *aexU*. All trees are rooted using *Aeromonas hydrophila* as an outgroup. Numbers represent the posterior probability for that split, edge lengths are scaled to average substitutions per site in the posterior distribution.
(PDF)

Figure S3 **Scatter plot of agreement of embedded quartets from inferred recombinant fragments with each phenotype grouping.** Each family is represented by a symbol with fragments labeled on the plot: *aexT*, square cross; *aexU*, diamond cross; *ascFG*, diamond; *ascV*, triangle; *chiA*, solid square; *dnaJ*, solid circle; *gyrB*, solid triangle; *recA*, solid diamond. Symbols correspond to phenotype groupings by color: leech colonization, green; growth on blood, red; LD_50_ in *G. mellonella*, blue. A symbol for a gene family in strong agreement with a particular grouping will have a x value close to 1, or a y value close to 1 if in strong disagreement. Those with poor phylogenetic signal will be close to the origin.
(TIF)
