# Supplementary material for: Complex Evolutionary History of the Aeromonas veronii Group Revealed by Host Interaction and DNA Sequence Data
Source: PLoS One. 2011 Feb 16;6(2):e16751. doi: 10.1371/journal.pone.0016751 (PMC3040217; doi:10.1371/journal.pone.0016751)
Supplement: Analysis S1 — Identification of Informative Sites. (DOCX) [file pone.0016751.s001.docx]

**Identification of Informative Sites**

**Evolutionary history by supermatrix of characters.** Corresponding to the splits networks (Figure 4), Bayesian trees were inferred (Figure 5) from concatenations of all sequences, housekeeping gene sequences (*chiA*, *gyrB*, *dnaJ*, and *recA*) and T3SS gene sequences (*aexT*, *aexU*, *ascFG*, and *ascV*) in which internal nodes do not represent ancestral states (Figure 5). Given the evidence in sections (i) and (ii) above, clusters can be caused by two processes: the combined molecular diversity at each loci being less for organisms within the cluster than with those outside the cluster; or by recombination within the cluster having a homogenizing effect on the molecular diversity. It is important to note that the topology does not explicitly describe a tree-like history: internal nodes do not represent ancestral states even with an assumed rooting. The same relationships are recovered as in the splits networks with a few notable exceptions. According to all sequences, AER28 and LMG13695 are grouped together in the splits tree (Figure 4A) with an indication of the degree of conflicting signal, while in the tree (Figure 5A) they are separated by the clade containing Hv241, Hv231 and Hv221. In figure 5A, AMC34 is on a relatively long branch, while in Figure 4A it is placed in a central polytomy.

The rooted Baysian evolutionary grouping based on a concatenation of housekeeping genes and the chitinase gene (Figure S2A) did not resolve *A. allosaccharophila* (strain LMG140549) or AMC24 (posterior probability 0.55) with respect to a resolved clade of the remaining AVG strains (posterior probability 0.97). The phylogenies of *aexU* inferred recombinant fragments 2 and 3 (Figure S2B and C) split the AVG group from *A. allosaccharophila* and AMC22 with a relatively long branch with strong support while fragment 1 (Figure S2D) was less well resolved but also placed *A. allosaccharophila* the furthest from the AVG group.

**Identification of informative sequences.** Table S3 shows the sequence alignments ranked by agreement score, which indicates the proportion of phylogenetic signal in each alignment that is consistent with the plurality consensus signal; Table S4 shows the alignments ranked by contribution score, which indicates the proportion of potential phylogenetic signal in each alignment that is consistent with the plurality consensus signal. Both tables include the information content score indicating how much that gene family sequence contributed to the overall signal (including disagreement with the plurality consensus). A comparison of the three scores for *gyrB* fragment 2 (between predicted recombination breakpoints; see Methods) shows that they have a low information score. The proportion of their signal that is in agreement with the plurality consensus is high (a high agreement score) and a consequence of the low information score has a relatively small influence on the consensus signal. In contrast, *aexU* fragment 2 contains a relatively strong signal and even though less than half of it is consistent with the plurality consensus, it makes a relatively high contribution.

The data set is characterized by conflicting signals between fragments indicated by a wide range of plurality consensus agreement scores and the non-treelike appearance of the splits network representations of the quartet decomposition plurality signals. There was not a clear dichotomy between the housekeeping and T3SS associated gene sequences with respect to any of the scoring criteria. Gene sequences associated with one class of protein function did not dominate the plurality consensus signal over the other.
