# Supplementary material for: Complex Evolutionary History of the Aeromonas veronii Group Revealed by Host Interaction and DNA Sequence Data
Source: PLoS One. 2011 Feb 16;6(2):e16751. doi: 10.1371/journal.pone.0016751 (PMC3040217; doi:10.1371/journal.pone.0016751)
Supplement: Table S1 — Summary of analyzed sequences. (DOC) [file pone.0016751.s002.doc]

Table S1. Summary of analyzed sequences.

| Product name *^a^* | | | Gene | No. of sequences | Aligned length | Polymorphisms | Informative sites | Global Identity *^b^* | CG*^c^* |
| --- | --- | --- | --- | --- | --- | --- | --- | --- | --- |
| Exoenzyme T | | | *aexT* | 20 | 399 | 15 | 6 | 96.24 | 57.33 |
| Exoenzyme U | | | *aexU* | 17 | 546 | 127 | 69 | 73.99 | 64.5 |
| fragment 1 | | | | 17 | 153 | 22 | 10 | 76.73 |  |
| fragment 2 | | | | 17 | 195 | 40 | 28 | 79.49 |  |
| fragment 3 | | | | 17 | 198 | 65 | 31 | 67.17 |  |
| T3SS | *ascF* | | | 20 | 180 | 23 | 22 | 87.22 | 51.64 |
| T3SS | *ascG* | | | 20 | 261 | 29 | 20 | 88.89 | 62.89 |
| T3SS | *ascV* | | | 20 | 489 | 48 | 36 | 90.18 | 64.34 |
| fragment 1 | | | | 20 | 162 | 14 | 12 | 91.36 |  |
| fragment 2 | | | | 20 | 111 | 9 | 8 | 91.89 |  |
| fragment 3 | | | | 20 | 216 | 25 | 16 | 88.43 |  |
| Chitinase A | *chiA* | | | 20 | 272 | 71 | 61 | 79.90 | 62.59 |
| J domain protein | *dnaJ* | | | 20 | 687 | 68 | 32 | 90.10 | 64.76 |
| fragment 1 | | | | 20 | 483 | 49 | 21 | 89.86 |  |
| fragment 2 | | | | 20 | 204 | 19 | 11 | 90.69 |  |
| DNA gyrase, subunit B | *gyrB* | | | 20 | 861 | 118 | 58 | 86.30 | 58.69 |
| fragment 1 | | | | 20 | 249 | 49 | 25 | 80.32 |  |
| fragment 2 | | | | 20 | 278 | 34 | 16 | 87.77 |  |
| fragment 3 | | | | 20 | 334 | 35 | 17 | 89.52 |  |
| Recombinase, subunit A | | *recA* | | 20 | 390 | 56 | 36 | 85.64 | 59.26 |

*^a^* The additional fragments of *aexU*, *ascV*, *dnaJ* and *gyrB* are between predicted recombination breakpoints.

*^b^* Global percent identity, which is the percentage of identical sites in the entire alignment.

*^c^* Percent GC content.
