# Supplementary material for: Complex Evolutionary History of the Aeromonas veronii Group Revealed by Host Interaction and DNA Sequence Data
Source: PLoS One. 2011 Feb 16;6(2):e16751. doi: 10.1371/journal.pone.0016751 (PMC3040217; doi:10.1371/journal.pone.0016751)
Supplement: Table S2 — Approximately Unbiased test. (DOC) [file pone.0016751.s003.doc]

Table S2. Approximately Unbiased test; p-values for maximum-likelihood tree topologies inferred from each gene alignment against a concatenation of all gene alignments.

|  | Topology set *^a^* | | |
| --- | --- | --- | --- |
| Topology sequence | 1 NNIs *^b^* | 2 NNIs | SPR *^c^* |
| *aexT* | 0 | 0 | 0 |
| *aexU* | 0.001 | 0 | 0 |
| *ascFG* | 0 | 0 | 0 |
| *ascG* | 0 | 0 | 0 |
| *ascV* | 0 | 0 | 0 |
| *chiA* | 0 | 0 | 0 |
| *dnaJ* | 0 | 0 | 0.001 |
| *gyrB* | 0.001 | 0.001 | 0 |
| *recA* | 0 | 0 | 0 |
| Concatenation | 0.725 | 0.981 | 0.972 |

*^a^* Three sets of topologies were tested, each containing 5 additional tree permutations per gene obtained by one or two random NNI moves or random SPR moves.

*^b^* Nearest neighbor interchange.

*^c^* Random subtree prune and regraft.
