# Supplementary material for: Complex Evolutionary History of the Aeromonas veronii Group Revealed by Host Interaction and DNA Sequence Data
Source: PLoS One. 2011 Feb 16;6(2):e16751. doi: 10.1371/journal.pone.0016751 (PMC3040217; doi:10.1371/journal.pone.0016751)
Supplement: Table S3 — Agreement and information content scores. (DOCX) [file pone.0016751.s004.docx]

Supplementary Table 3. Agreement and information content scores.

| All sequences | | | Housekeeping gene sequences | | | T3SS gene sequences | | |
| --- | --- | --- | --- | --- | --- | --- | --- | --- |
| Fragment | Agreement*^a^* | Information*^b^* | Fragment | Agreement | Information | Fragment | Agreement | Information |
| *gyrB* 2 | 0.83 | 0.04 | *gyrB* 3 | 0.84 | 0.17 | *aexT* | 0.87 | 0.07 |
| *gyrB* 3 | 0.76 | 0.17 | *gyrB* 2 | 0.79 | 0.04 | *aexU* 3 | 0.86 | 0.08 |
| *aexT* | 0.68 | 0.08 | *gyrB* 1 | 0.67 | 0.22 | *ascV* 1 | 0.81 | 0.06 |
| *ascV* 1 | 0.66 | 0.06 | *dnaJ* 1 | 0.64 | 0.16 | *ascFG* 2 | 0.71 | 0.18 |
| *gyrB* 1 | 0.64 | 0.22 | *recA* | 0.60 | 0.26 | *ascV* 3 | 0.64 | 0.10 |
| *dnaJ* 1 | 0.62 | 0.14 | *chiA* | 0.57 | 0.34 | *aexU* 2 | 0.60 | 0.37 |
| *ascFG* 2 | 0.61 | 0.20 | *dnaJ* 2 | 0.51 | 0.15 | *ascFG* 1 | 0.56 | 0.23 |
| *recA* | 0.57 | 0.26 |  |  |  | *aexU* 1 | 0.44 | 0.02 |
| *aexU* 3 | 0.57 | 0.07 |  |  |  | *ascV* 2 | 0.39 | 0.17 |
| *chiA* | 0.57 | 0.34 |  |  |  |  |  |  |
| *ascV* 3 | 0.57 | 0.10 |  |  |  |  |  |  |
| *ascFG* 1 | 0.53 | 0.27 |  |  |  |  |  |  |
| *dnaJ* 2 | 0.53 | 0.15 |  |  |  |  |  |  |
| *aexU* 2 | 0.49 | 0.38 |  |  |  |  |  |  |
| *aexU* 1 | 0.45 | 0.03 |  |  |  |  |  |  |
| *ascV* 2 | 0.45 | 0.20 |  |  |  |  |  |  |

*^a^* Agreement scores indicating the proportion of phylogenetic signal in each alignment that is consistent with the plurality consensus signal inferred from quartet decomposition analysis where the maximum score of 1 indicates all embedded quartets with sufficient resolution (*i.e.*, the signal above the noise) were consistent.

*^b^* The information content score indicates the proportion of embedded quartets making up the signal, including those which are in conflict with the plurality consensus signal.
