# Supplementary material for: Complex Evolutionary History of the Aeromonas veronii Group Revealed by Host Interaction and DNA Sequence Data
Source: PLoS One. 2011 Feb 16;6(2):e16751. doi: 10.1371/journal.pone.0016751 (PMC3040217; doi:10.1371/journal.pone.0016751)
Supplement: Table S4 — Contribution and information content scores. (DOCX) [file pone.0016751.s005.docx]

Supplementary Table 4. Contribution and information content scores.

| All sequences | | | Housekeeping gene sequences | | | T3SS gene sequences | | |
| --- | --- | --- | --- | --- | --- | --- | --- | --- |
| Fragment | Contribution*^a^* | Information*^b^* | Fragment | Contribution | Information | Fragment | Contribution | Information |
| *chiA* | 0.204 | 0.335 | *chiA* | 0.202 | 0.335 | *aexU* 2 | 0.245 | 0.378 |
| *aexU* 2 | 0.203 | 0.378 | *recA* | 0.192 | 0.259 | *ascFG* 2 | 0.147 | 0.200 |
| *recA* | 0.191 | 0.259 | *gyrB* 1 | 0.168 | 0.220 | *ascFG* 1 | 0.138 | 0.273 |
| *gyrB* 1 | 0.159 | 0.220 | *gyrB* 3 | 0.149 | 0.166 | *ascV* 2 | 0.077 | 0.198 |
| *ascFG* 1 | 0.153 | 0.273 | *dnaJ* 1 | 0.115 | 0.142 | *ascV* 3 | 0.072 | 0.101 |
| *ascFG* 2 | 0.141 | 0.200 | *dnaJ* 2 | 0.091 | 0.154 | *aexU* 3 | 0.070 | 0.066 |
| *gyrB* 3 | 0.139 | 0.166 | *gyrB* 2 | 0.033 | 0.040 | *aexT* | 0.067 | 0.077 |
| *ascV* 2 | 0.102 | 0.198 |  |  |  | *ascV* 1 | 0.046 | 0.061 |
| *dnaJ* 1 | 0.102 | 0.142 |  |  |  | *aexU* 1 | 0.014 | 0.029 |
| *dnaJ* 2 | 0.091 | 0.154 |  |  |  |  |  |  |
| *ascV* 3 | 0.063 | 0.101 |  |  |  |  |  |  |
| *aexT* | 0.054 | 0.077 |  |  |  |  |  |  |
| *ascV* 1 | 0.041 | 0.061 |  |  |  |  |  |  |
| *aexU* 3 | 0.038 | 0.066 |  |  |  |  |  |  |
| *gyrB* 2 | 0.036 | 0.040 |  |  |  |  |  |  |
| *aexU* 1 | 0.017 | 0.029 |  |  |  |  |  |  |

*^a^*The Contribution score indicates the proportion of potential phylogenetic signal in each alignment that is consistent with the plurality consensus signal inferred from quartet decomposition analysis where the maximum score of 1 indicateds all embedded quartets were sufficiently resolved (*i.e.*, all signal and no noise) and were consistent with the plurality.

*^b^* The information content score indicates the proportion of embedded quartets making up the signal, including those which are in conflict with the plurality consensus signal.
