# Supplementary figures and images for: Complex Evolutionary History of the Aeromonas veronii Group Revealed by Host Interaction and DNA Sequence Data
Source: PLoS One. 2011 Feb 16;6(2):e16751. doi: 10.1371/journal.pone.0016751 (PMC3040217; doi:10.1371/journal.pone.0016751)

A

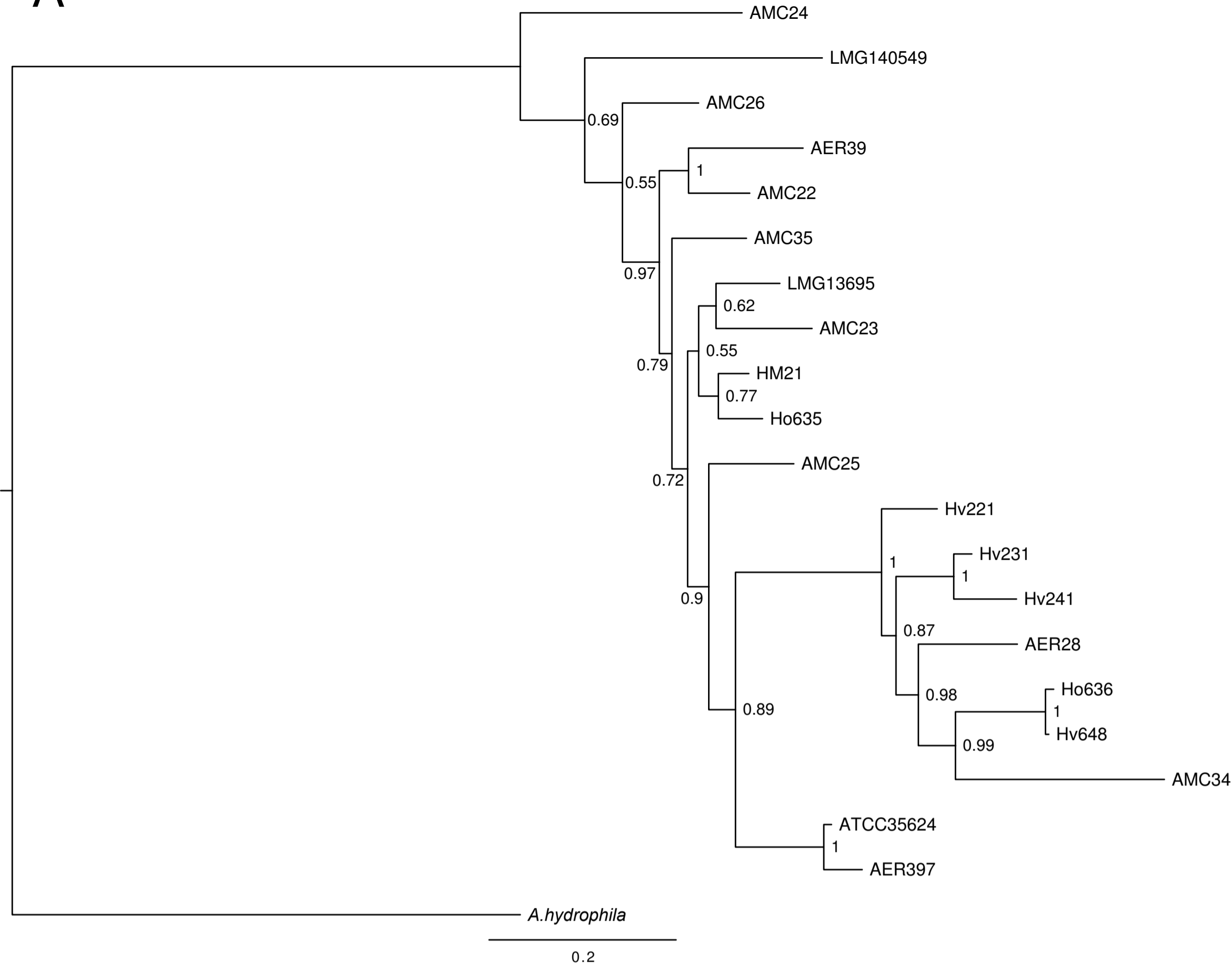

B

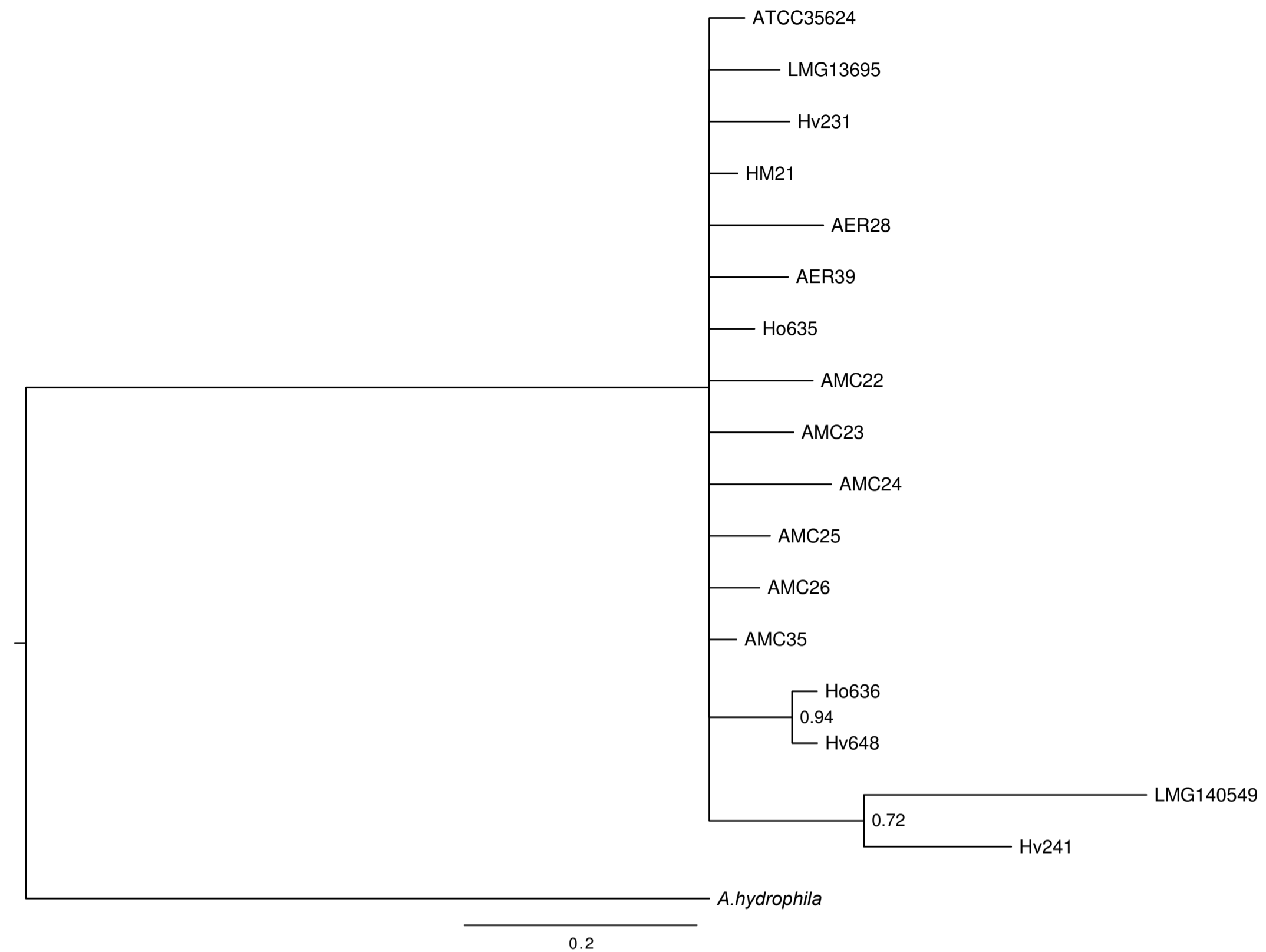

C

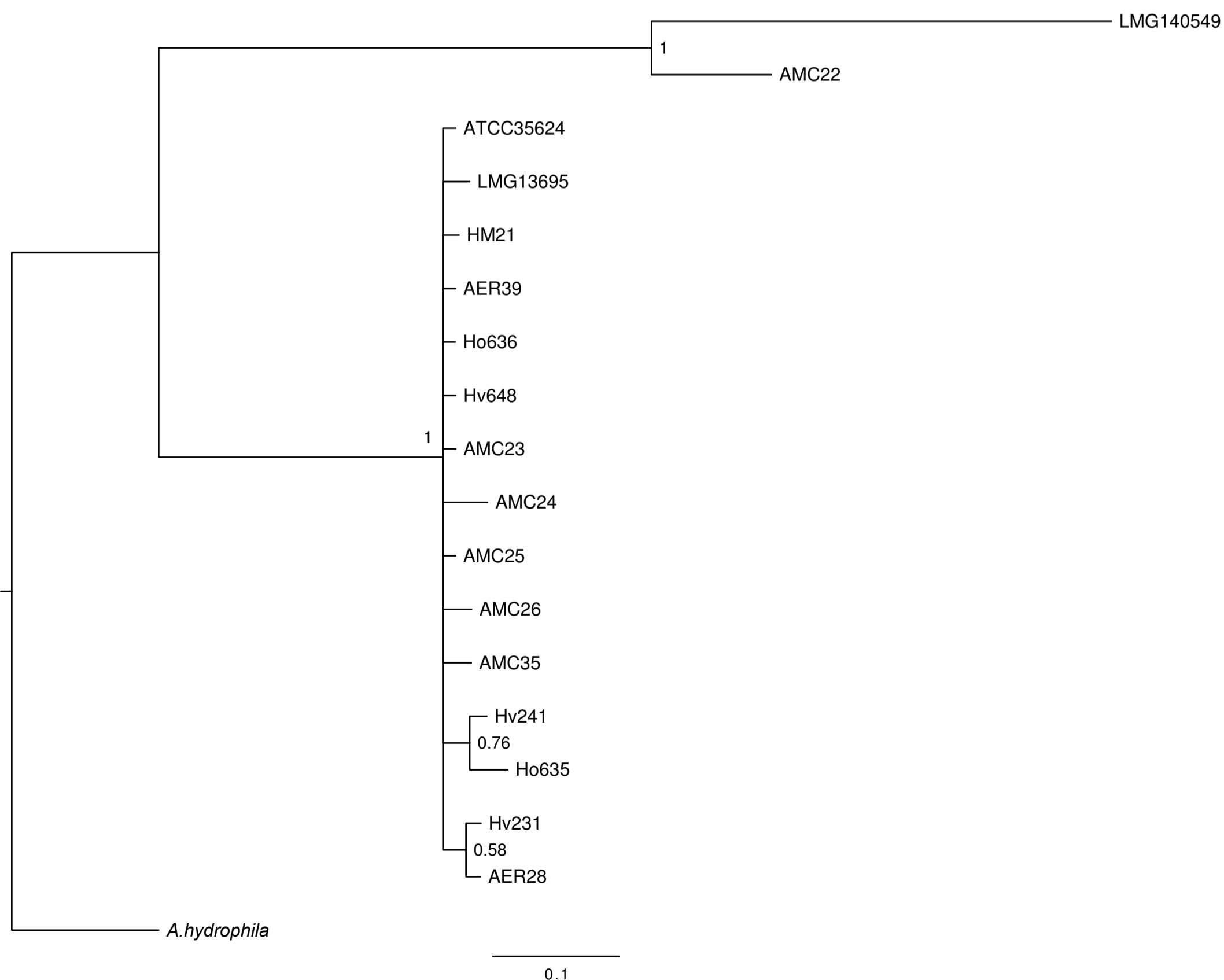

D

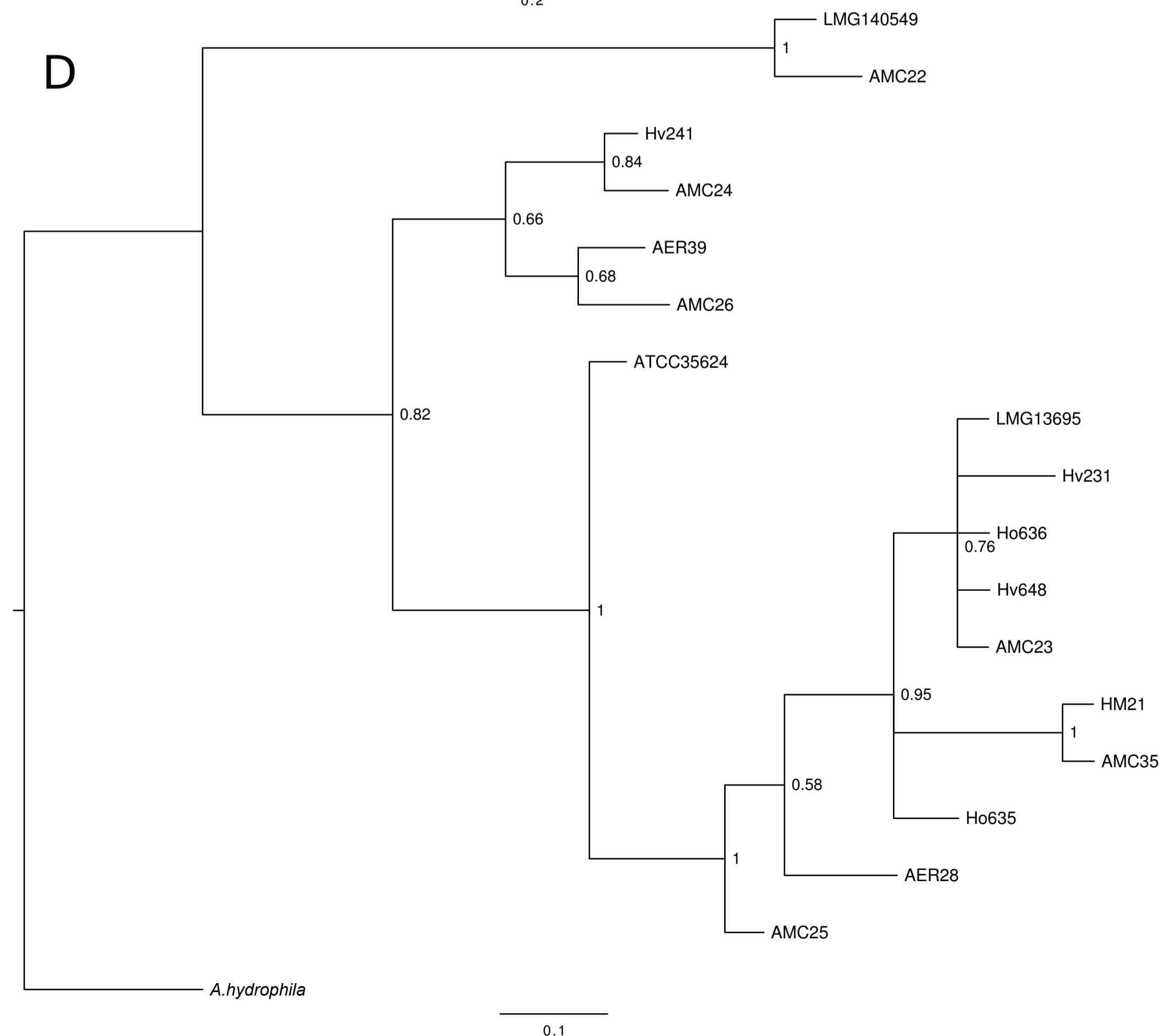

Supplement: Figure S1 — Split networks and Bayesian trees inferred from concatenations gene family alignments. A. These evolutionary relationships were inferred from maximum likelihood trees of 100 bootstrap replicates, weighted by frequency, of chiA, gyrB, dnaJ, recA, aexT, aexU, ascFG, and ascV. Alignments were divided at predicted recombination breakpoints determined by distinct evolutionary histories. Edge lengths are scaled to the number of embedded quartets in agreement with that split and split widths represent a departure from a tree-like signal. In the case of Aeromonas veronii group strains splits with width are likely to be indicative of recombination across that split. B. From the same concatenated sequences Bayesian trees were inferred. Numbers represent the posterior probability for that split, edge lengths are scaled to average substitutions per site in the posterior distribution. (PDF) [file pone.0016751.s006.pdf]

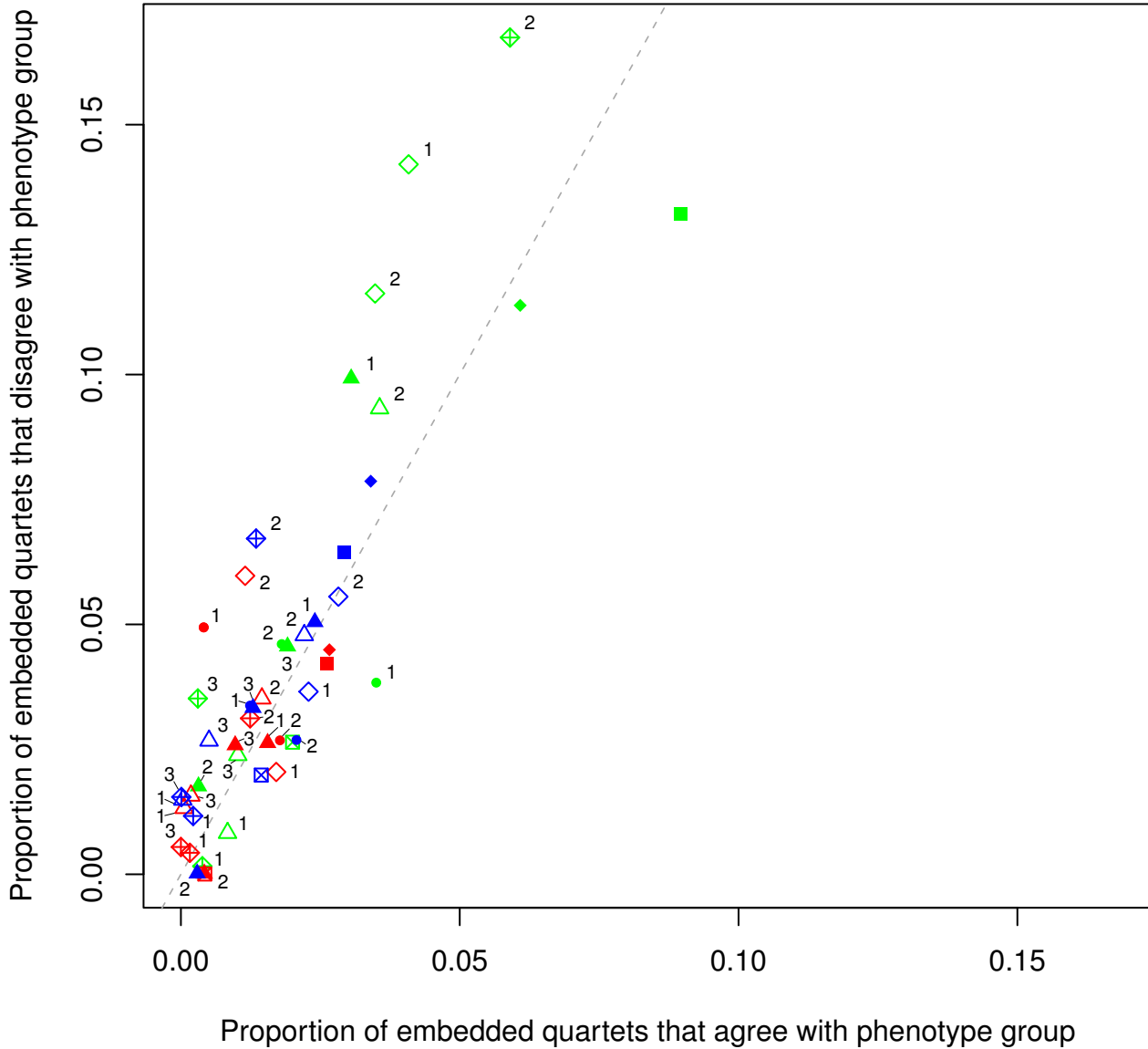

Supplement: Figure S2 — Bayesian trees inferred from housekeeping genes and each aexU recombinant fragment. (A). Tree inferred from concatenation of housekeeping gene sequences (chiA, gyrB, dnaJ, and recA); (B, C and D) trees inferred from each of the three inferred recombinant fragments of aexU. All trees are rooted using Aeromonas hydrophila as an outgroup. Numbers represent the posterior probability for that split, edge lengths are scaled to average substitutions per site in the posterior distribution. (PDF) [file pone.0016751.s007.pdf]

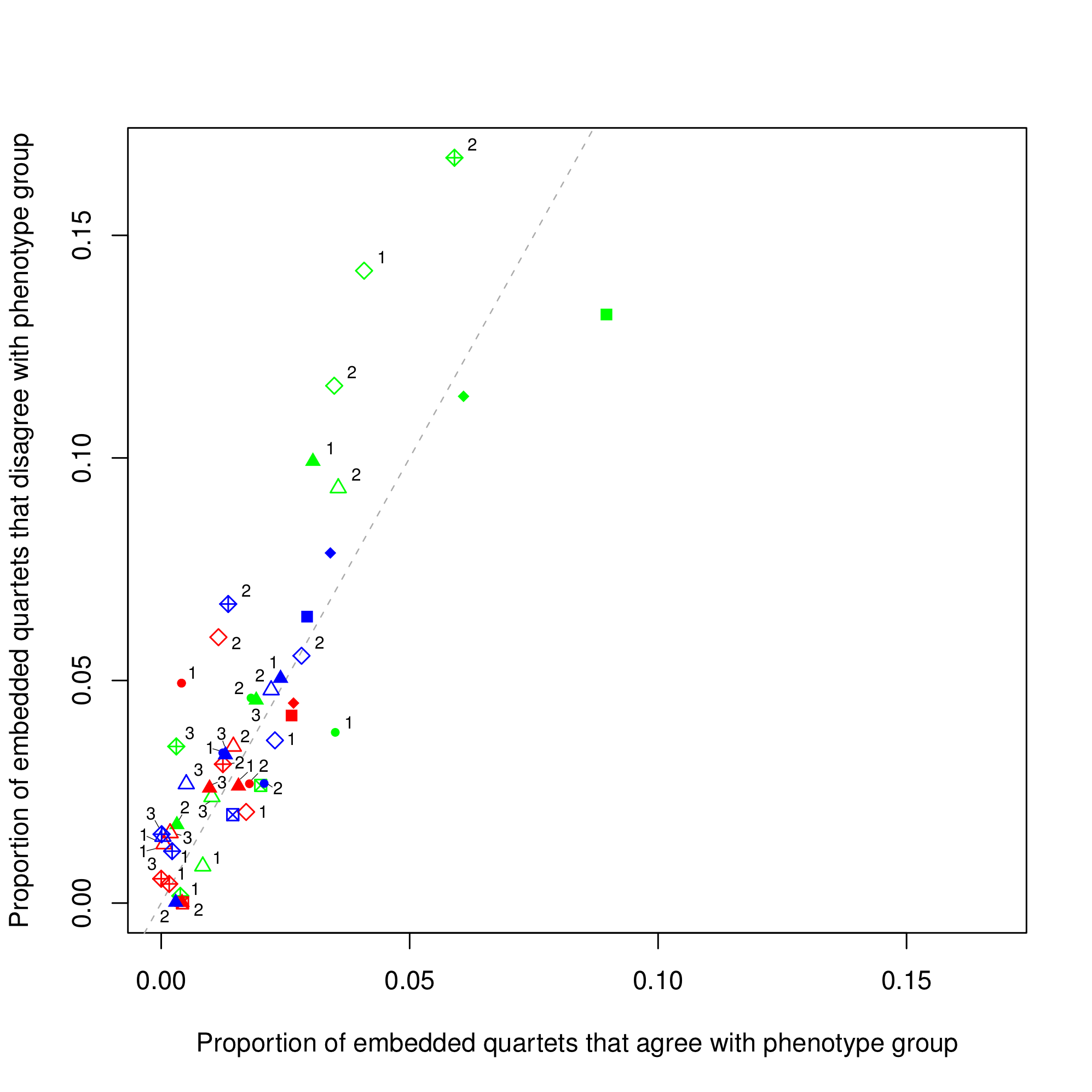

Supplement: Figure S3 — Scatter plot of agreement of embedded quartets from inferred recombinant fragments with each phenotype grouping. Each family is represented by a symbol with fragments labeled on the plot: aexT, square cross; aexU, diamond cross; ascFG, diamond; ascV, triangle; chiA, solid square; dnaJ, solid circle; gyrB, solid triangle; recA, solid diamond. Symbols correspond to phenotype groupings by color: leech colonization, green; growth on blood, red; LD50 in G. mellonella, blue. A symbol for a gene family in strong agreement with a particular grouping will have a x value close to 1, or a y value close to 1 if in strong disagreement. Those with poor phylogenetic signal will be close to the origin. (TIF) [file pone.0016751.s008.tif]
